# Supplementary figures and images for: Activation of Shigella flexneri type 3 secretion requires a host-induced conformational change to the translocon pore
Source: PLoS Pathog. 2019 Nov 14;15(11):e1007928. doi: 10.1371/journal.ppat.1007928 (PMC6879154; doi:10.1371/journal.ppat.1007928)

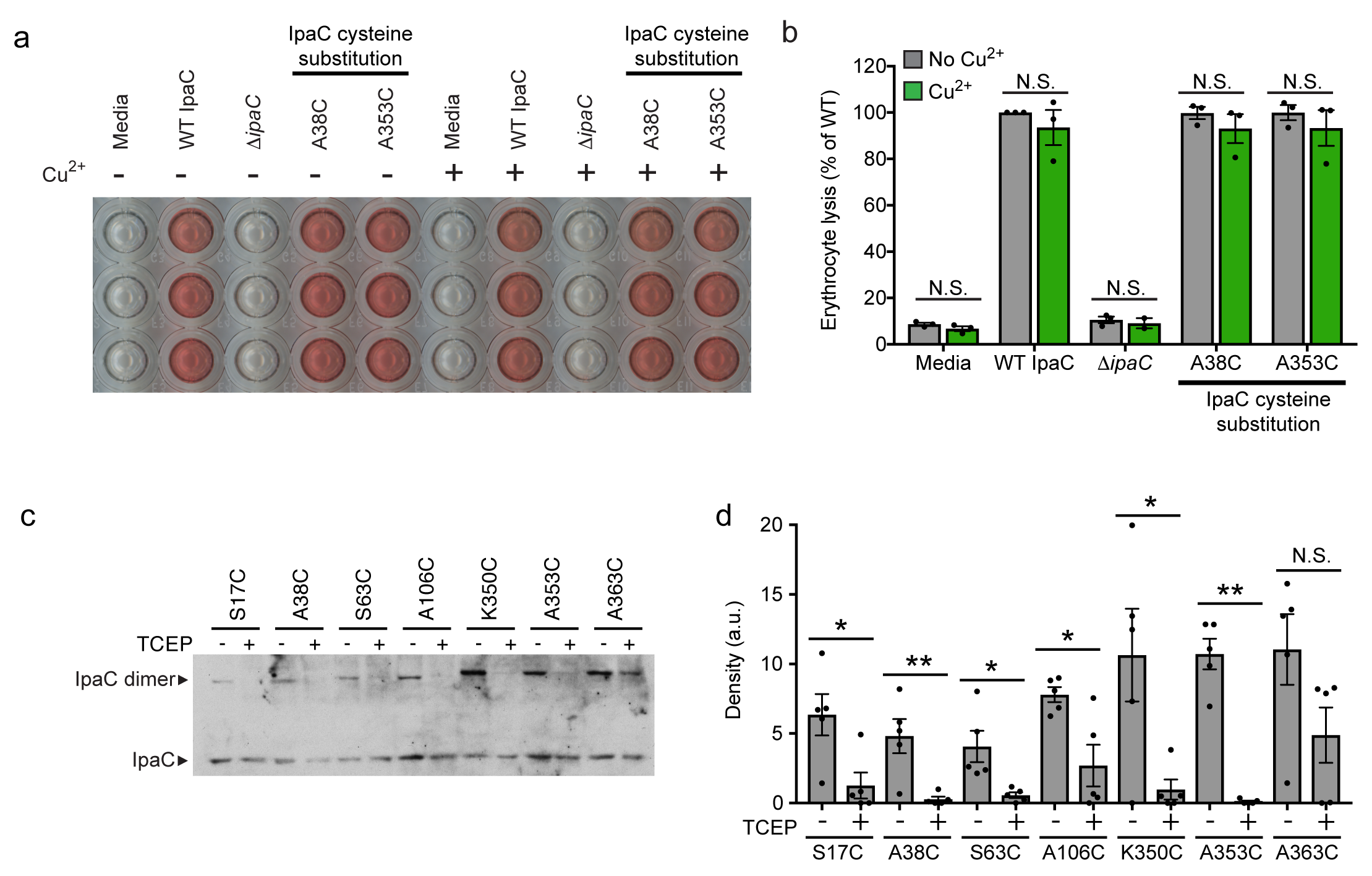

Supplement: S1 Fig — (a-b) Copper does not inhibit the formation of translocon pores. Erythrocyte lysis as a result of pore formation in cell membranes during co-culture of erythrocytes with S. flexneri. S. flexneri ΔipaC strains producing cysteine substitution derivatives of IpaC in the presence or absence of copper. Representative image of released hemoglobin in the supernatants of co-cultured erythrocytes (a). Efficiency of erythrocyte lysis, as a function of the abundance of hemoglobin in the co-culture supernatants, quantified by A570 (b). (c-d) Gel shift of IpaC dimer bands following exposure to copper is diminished by addition of the membrane-impermeant reductant TCEP. Representative western blot of IpaC (c). Densitometry of the slow migrating bands (d). Graphed data are presented as mean ± SEM of three or more independent experiments. *, p<0.05; **, p<0.01; N.S., not significant. One-way ANOVA with Dunnett’s post hoc test (b). Student’s t-test (d). (TIF) [file ppat.1007928.s001.tif]

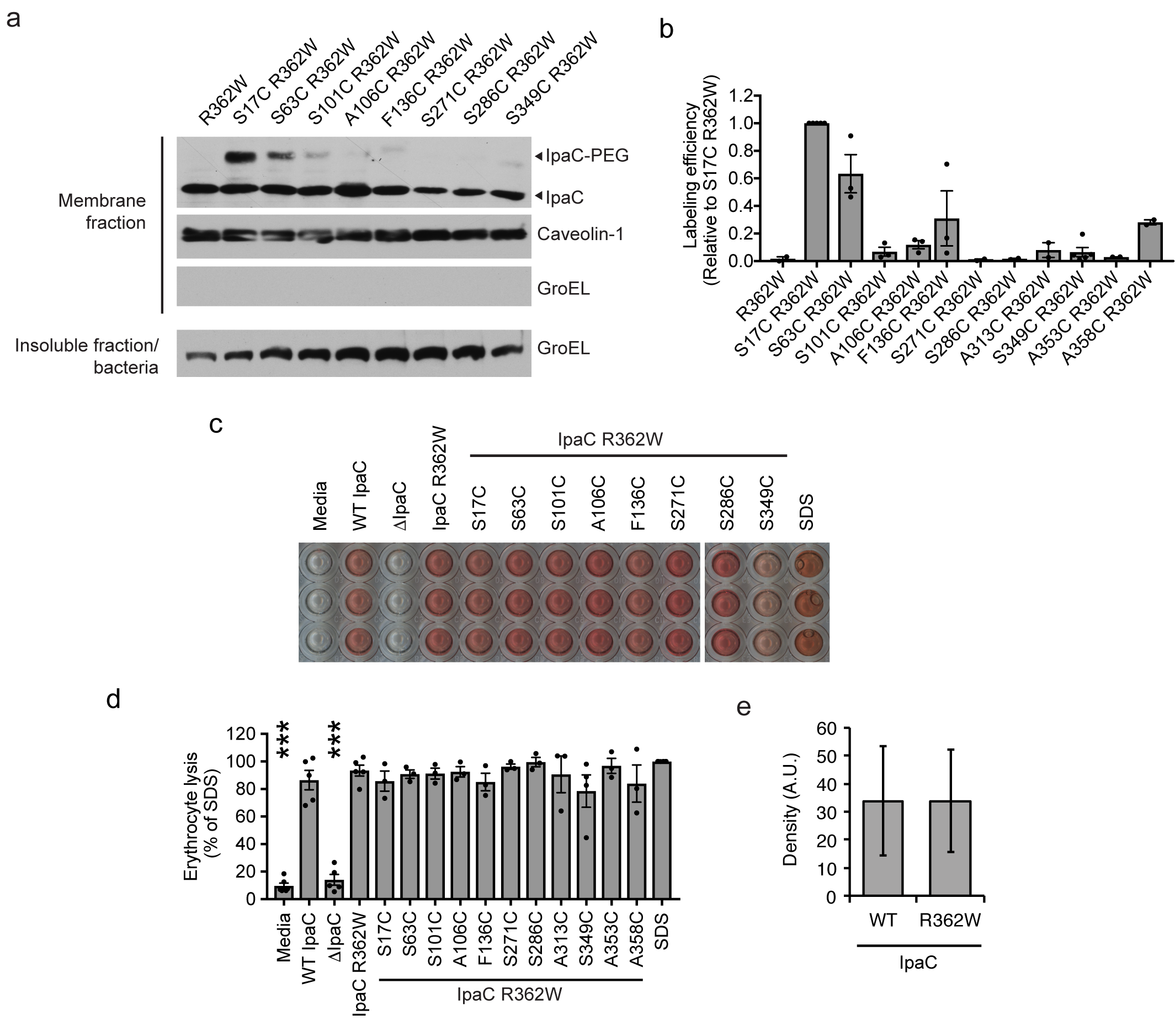

Supplement: S2 Fig — Accessibility of membrane-embedded IpaC R362W to labeling with PEG5000-maleimide upon infection of HeLa cells with S. flexneri producing the indicated single cysteine substitution derivatives of IpaC R362W. (a) Gel shift of PEG5000-maleimide labeled IpaC in the plasma membrane-enriched fraction of infected HeLa cells. Representative western blot of IpaC. IpaC-PEG5000, IpaC R362W derivatives labeled with PEG5000-maleimide; IpaC, unlabeled IpaC R362W derivatives; caveolin-1, plasma membrane protein; GroEL, bacterial cytosolic protein. (b) Relative accessibility of IpaC R362W cysteine substitutions. Densitometry analysis of IpaC-PEG5000 bands from experiments represented in panel a. Two independent experiments; mean ± SEM. (c-d) Efficiency of pore formation in mammalian membranes as measured by erythrocyte lysis during co-culture of erythrocytes with S. flexneri ΔipaC strains producing the indicated cysteine substitution derivatives of IpaC R362W. (c) Representative images of hemoglobin released into the supernatants of co-cultured erythrocytes. (d) Efficiency of erythrocyte lysis, as a function of the abundance of hemoglobin in the co-culture supernatants, quantified by A570 in experiments represented in panel c. Three independent experiments for each cysteine mutant; mean ± SEM. Strains producing an IpaC R362W cysteine substitution were not statistically different from the strain producing IpaC R362W. ***, p <0.001. Two-way ANOVA with a Dunnett’s post hoc test. (e) IpaC R362W is inserted in mammalian membranes at an efficiency similar to that of WT IpaC. The abundance of WT IpaC and IpaC R362W in the membrane-enriched fractions of Vim+/+ MEFs. Mean ± SEM from three independent experiments. No significant difference between means (Student’s t-test). (TIF) [file ppat.1007928.s002.tif]

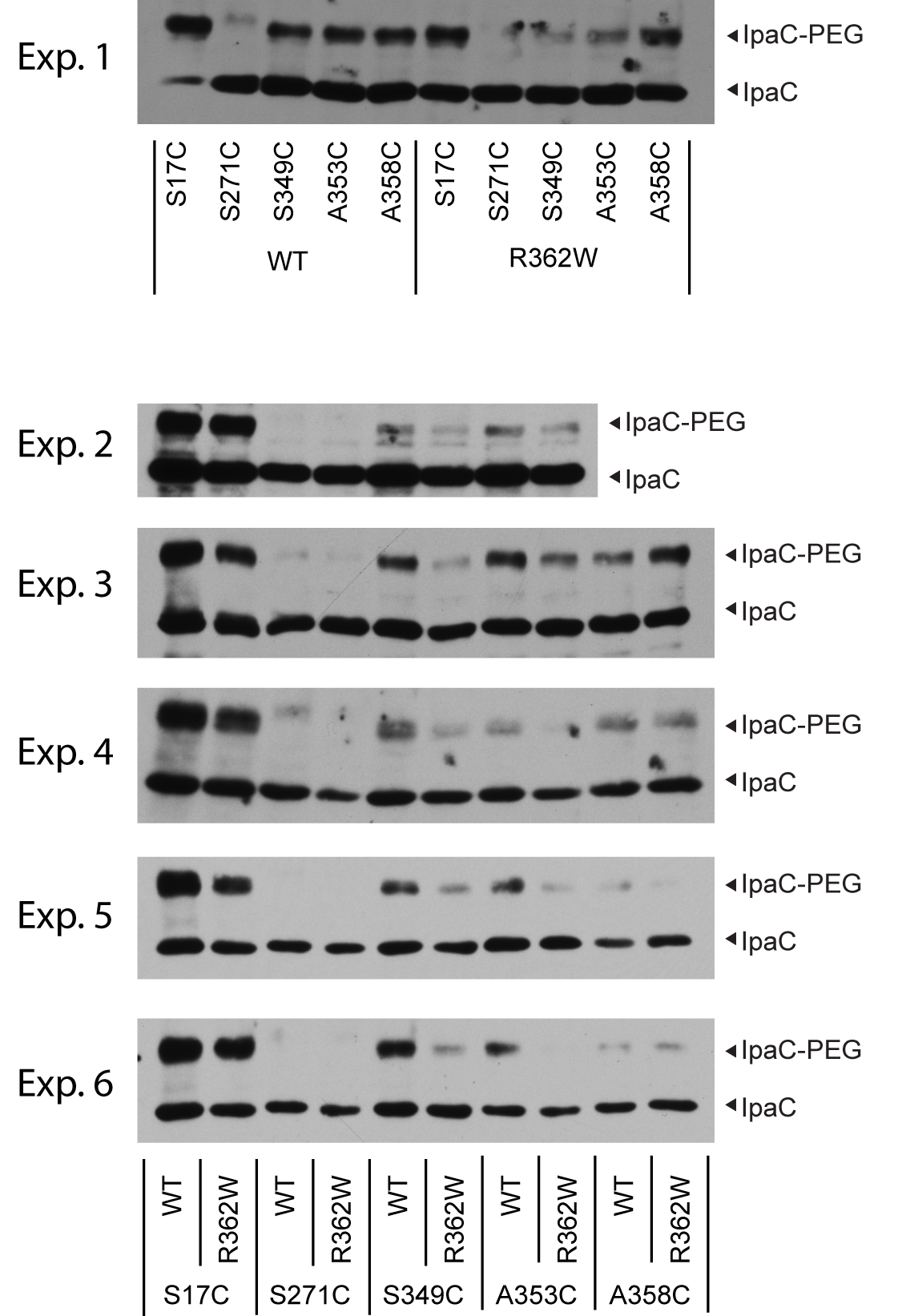

Supplement: S3 Fig — PEG5000-maleimide labeling of sulfhydryl groups in cysteine substitution derivatives in the context of WT IpaC or IpaC R362W during S. flexneri infection of HeLa cells. Western blots from each of six independent experiments performed. (TIF) [file ppat.1007928.s003.tif]

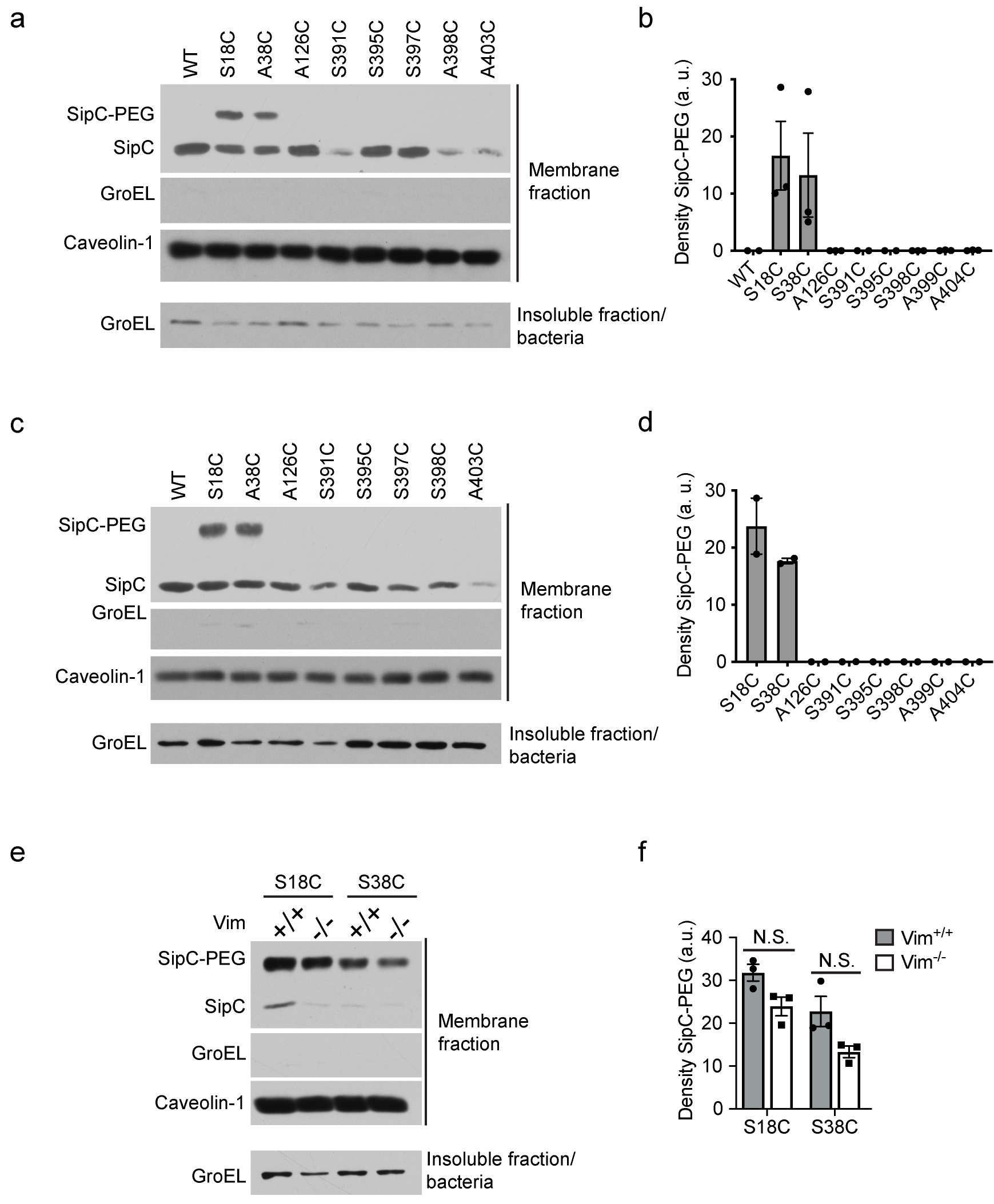

Supplement: S4 Fig — PEG5000-maleimide labeling of sulfhydryl groups in cysteine substitution derivatives in the context of WT SipC during S. Typhimirium infection of Vim+/+ and Vim-/- cells. (a-d) Representative western blots of PEG5000-maleimide labeling of SipC cysteine derivates in membrane-enriched fractions from Vim+/+ (a) and Vim-/- (c) MEFs. Densitometry analysis of bands corresponding to SipC-PEG5000 from (a) and (c), respectively (b and d). (e-f) Direct comparison of PEG5000-maleimide labeling of SipC upon infection of Vim+/+ and Vim-/- MEFs with S. Typhimurium ΔsipC producing S18C or A38C. (e) Representative western blots. (f) Densitometry analysis of bands corresponding to SipC-PEG5000 from (e). SipC-PEG, SipC derivatives labeled with PEG5000-maleimide; SipC, unlabeled SipC derivatives; Caveolin-1, marker of eukaryotic plasma membrane; GroEL, bacterial cytosolic protein. Graphed data are presented as mean ± SEM of two (c-d) or three (a-b and e-f) independent experiments. N.S., not significant. Two-way ANOVA with Sidak post hoc test. (TIF) [file ppat.1007928.s004.tif]
