# Supplementary material for: Activation of Shigella flexneri type 3 secretion requires a host-induced conformational change to the translocon pore
Source: PLoS Pathog. 2019 Nov 14;15(11):e1007928. doi: 10.1371/journal.ppat.1007928 (PMC6879154; doi:10.1371/journal.ppat.1007928)
Supplement: Attachment — Submitted filename: Response to reviewers.docx [file ppat.1007928.s005.docx]

**Detailed response to Editor’s and reviewer’s comments**

We thank the reviewers for their constructive criticisms of our manuscript and believe that by responding to these comments, the manuscript is much improved.

Response to the Editor’s comments:

All reviewers got consensus that this work should advance our understanding of the role of IpaC during host cell invasion.

Reviewers raised the question whether the conformational change found in IpaC is a general mechanism of T3SS-host interaction. It would be nice if you could test if translocons formed by IpaC-orthologuous (from Pseudomonas or Salmonella) do also change their conformation upon host contact.

**We agree with the reviewers that investigation of whether orthologous translocon proteins undergo conformational changes upon host interaction is interesting. Like IpaC, the *S.* Typhimurium translocon protein SipC (a homolog of IpaC) interacts with Intermediate filaments (Russo BC, et al., Nat. Microbiol. 2016). We tested whether intermediate filaments alter the conformation of SipC (Fig S4) by analyzing the extracellular accessibility of residues in SipC that are homologous to those IpaC residues that displayed intermediate filament-dependent changes in extracellular accessibility (Fig 3). Thus, using the methodology we used for IpaC, we are unable to detect intermediate filament-dependent conformational changes in SipC. These data are included in the revised manuscript (new Fig S4 and lines 300-317).**

Response to the Reviewers’ Questions:

**Part I - Summary**
Please use this section to discuss strengths/weaknesses of study, novelty/significance, general execution and scholarship.

Reviewer #1:

Reviewer #1: The presented experiments are well designed and well conducted, controls thoughtfully planned and extensively performed, tools validated in previous papers, experiments allow clear cut conclusion. The authors use MEF cells which is a logical choice, since they found several IFs to be involved in IpaC interaction (Russo et al 16), and in MEFs vimentin Kos are available.

It would have been nice if authors would have explored a bit more the open directions. This could have been either (i) on a more mechanistical level with a biophysical approach, or (ii) to explore general meaning in host-pathogen interactions of T3SSs with host IFs in comparison with other pathogens.

- Authors did not generate new tools, all was published and used already by them or others.
- With regard to research: Several teams hypothesized that a conformational change initiates secretion. It has been shown already by other team that the T3SS IpaC homolog PopD undergoes conformational change required for secretion. It has been unknown what triggers secretion. The Goldberg team previously showed that IpaC-IF interactions after translocon formation is required for stable docking and thus continuing secretion. They proposed this interaction induces conformational change. Here they show that the trigger is their described IF-IpaC interaction with a singular experimental strategy.

I would have appreciated if the authors developed this further, to verify this with other techniques. Or to explore further the mechanistic consequences, compare different T3SS of different pathogens etc.

**We appreciate the reviewer’s suggestion to further investigate the generalizability of the presented phenotypes. We tested and now include analysis of whether there is an intermediate filament-dependent conformational change in the *S.* Typhimurium translocon pore protein SipC (Fig S4 and response to Editor, above).**

Reviewer #2: The manuscript by Russo et al. presents one of the most detailed views to date of docking between the type III secretion needle tip, and the translocation pore. The authors use disulfide bonds introduced into one of the pore-forming translocator proteins, IpaC, to demonstrate that tethering of translocator proteins can specifically interfere with this docking step. Moreover, the authors demonstrate by assaying accessibility of cysteine residues engineered into IpaC, that binding of IpaC to intermediate filaments, a critical step for docking, induces a conformational change. Taken together, these data indicate that binding of IpaC to intermediate filament proteins induces a conformational change in the translocation pore, which then facilitates docking of the needle-tip to the pore. The authors also present preliminary data that argues that triggering of effector secretion involves a discrete conformational step that follows docking, but the data for this are weaker. Taken together, the work presented here represents a significant step forward in our understanding of translocon function, which is a critical component of this important virulence factor.

Reviewer #3: General comments

The manuscript builds on previous work from the group on IpaC, a component of the translocon of the T3SS of Shigella. The authors have introduced cysteine residues into the protein and examined their ability to be cross linked in the presence/absence of copper to act as an oxidant. The effect of the membrane-impermeable reductant TCEP was also assessed. The work relies on changing residues in a protein for which folding is critical, in combination with calcium/TCEP to infer changes in accessibility of residues, conformation, and thence infer these changes could lead to changes in secretion by transducing a signal via a structural alteration down the T3SS needle to the cytoplasm where secretion hierarchy is determined.

The work is well written, and results clearly presented. I have provisos about the overall approaches, and how much weight can be placed on the results, but IpaC and related proteins are difficult to work with at a structural level, so the approaches taken by the authors are reasonable given the challenges of understanding the how the translocon works at the atomic level. However could they confirm their findings using other chemicals with similar or mismatched properties to Ca and TCEP?

**We agree with the reviewer that complementary approaches to address biological questions helps to confirm findings. We chose copper-based methods specifically because copper is widely used to induce disulfide bonds in biological systems and has relatively low toxicity. Indeed, we tested multiple copper derivatives, with similar experimental outcomes. As shown below, different versions of copper result in a similar efficiency of disulfide bond formation. Although in all cases, it is the copper moiety of each derivative that catalyzes disulfide bond formation, the three copper derivatives retain distinct chemical features.**

**In addition, we discussed non-copper alternatives with a chemist, who recommended testing DMSO. With DMSO, we observed non-specific effects on *Shigella* infection at various concentrations and chose to not pursue additional orthogonal approaches.**

**Part II – Major Issues: Key Experiments Required for Acceptance**

Reviewer #1: 1. Is the IF-IpaC conformational change a general T3SS-host interaction mechanism for effector secretion?
A. Comparison with P. aeruginosa: Are IFs like vimentin involved in the conformational changes described for the IpaC-homolog? Infect their Vim -/- cell line with P. aeruginosa and the described PopD mutants (is it possible that this is the missing triggering factor in the 2016-P. aeruginosa-publication the IF-PopD interaction?)
B. Or: Comparison with Salmonella: Both IpaC and SipC get activated via IFs (Russo…Goldberg 2016). In Russo et al 2019 they showed that the IpaC equivalent positions at the N-terminus of SipC are extracellular and accessible for modification; they have established SipC mutants (S18C, A38C). Here, they showed that in Shigella these mutants are crosslinked and useful tool to monitor conformational IpaC changes during docking and injection (Fig. 2, 3, 4). Can they show a conformational, vimentin-dependent change during Salmonella T3SS injection? What are differences?

**Please see response to Editor’s comments (above).**

C. They previously found orientation of the last C-terminal aa to lie inside the pore accessible from the extracelluar space, but not for Salmonella (Russo et al 2019). What happens when the last aa of the C-terminus of Salmonella SipC is changed to the IpaC sequence? Is there also a conformational change that involves SipC-IF interaction?

**Since the last seven amino acids of IpaC and SipC (including IpaC R362 and the analogous SipC residue, R407) are identical, we have not generated or tested a protein that is chimeric at the C-terminus. We hypothesize that other regions of IpaC or other aspects of the T3SS contribute to the differences in PEG5000 labeling near the C-terminus of IpaC and SipC. Further investigation of these differences is outside of the scope of this report.**

2. The C-terminus has been implied in actin remodeling via Src kinase, exactly the same region that they found to be involved in vimentin binding. Src phosphorylation of vimentin has been described that leads to vimentin disassembly (Oncogene 2019). Can they discuss this with regard to their model?

**We agree with the reviewer that this topic is important and have expanded the Discussion to include analysis of the relevance of these findings (lines 391-408).**

Reviewer #2: Line 287 last section re: Fig. 4, the decrease in TSAR for A38C is from 80% to 60% in 2 independent experiments. The defect in TSAR is minor, and two biological replicates is too few to really make a strong statement here. Couldn’t any short delay in docking account for this defect? After all, we don’t know if partial oxidation of IpaC in the pore prevents or delays docking. The fundamental question, does triggering of effector secretion derive from docking (an “induced fit” type model of triggering), or does it require a secondary conformational change, is interesting. The Fig. 4 data may argue toward the latter, but I don’t think the data are strong enough to make that call. I think the competing models and the data should be discussed, with some caveats, in the discussion, since this is a useful conversation for the field.

**We have added discussion about the activation of T3SS secretion (lines 383-408).**

If the authors leave the Fig. 4 data in, then I think they will need an additional biological replicate for the A38C TSAR data (Fig. 4C), since this is the crux of the figure, and 2 seems too few, particularly with the modest effect of adding the copper after spinning down the bacteria.

**We agree with the reviewer. We performed additional experiments to more closely examine whether there is a difference in the efficiency of TSAR activation for A38C (Fig. 4c).**

Reviewer #3: Line 189, conclusion correct? The reduction in docking in Vim-/- cells is substantial, and it is not certain whether they can conclude that calcium is having no effect, and there is very little docking in the first place.

**We appreciate that the low levels of docking indicate a need for additional sensitivity of the assay in Vim^-/-^ cells. Because of this, in each independent experiment, we analyzed 9 fields per condition; each condition totaled >50 cells for each experimental replicate (which corresponds to dozens of docked bacteria for each condition in each experiment involving Vim^-/-^ cells). If copper-induced crosslinking were to have a 3-5-fold (or lower) effect on docking under these conditions, we should have been able to detect it. For Vim^-/-^ cells, not only was docking similar in the presence and absence of copper, but no trend was observed that might suggest that copper was inhibiting docking to Vim^-/-^ cells.**

**However, from these experiments, we cannot rule out the possibility that the efficiency of bacterial docking to Vim^-/-^ cells is at background levels, in which case, it remains possible that disulfide bond formation at either A38C or A353C might non-specifically inhibit docking. Yet, the PEG labeling in Fig. 3 shows that the accessibility of IpaC residues change in an intermediate filament-dependent manner, which together with the copper crosslinking results, clearly demonstrate that a conformational change occurs in the pore and that this conformation change is dependent upon the interaction of intermediate filaments with IpaC.**

**We reworded the paragraph that includes line 189 (now line 201) to reflect these subtleties (lines 204-207).**

3E/F is a critical experiment, and quantification is not obvious from the blots provided. The authors should include all blots that were used to generate these data.

**As requested, the blots from the six independent experiments are now included (Fig. S3).**

**Part III – Minor Issues: Editorial and Data Presentation Modifications**
Please use this section for editorial suggestions as well as relatively minor modifications of existing data that would enhance clarity.


Reviewer #1: • Fig 1 as supplementary figure that only shows that crosslinking of cysteine substitution mutants (that have been already described in previous paper Russo et al 2019 to be accessible and able to modify) actually works. Quantification in (d) contains mutants L117C and S117C, where neither the WB are shown in (c), nor are they mentioned at all in the text.

**We now include mention of L117C and S117C in the text (line 145). Of note, analysis of these residues was not relevant for the subsequent experimental approaches or the conclusions of the paper.**

Since authors explain that only extracellular accessible residues can be modified (which they discovered already in Russo et al 2019), they show non-crosslinked, cytosolic residues again in Fig. 1 that are neither further investigated, nor discussed in the paper (S101C, S103C, S108C, A119C, F136C, A313C). In addition, authors perform in Fig. 3 experiments with modified residues 271, 349, and 358, but do also not include them here in their crosslinking control. Since they always do controls in WBs of single experiments, actually this figure is not needed anyway.

**We now include mention of S101C, S103C, S108C, A119C, F136C, A313C in the text (lines 144-147) and believe that these serve as important controls for those IpaC residues that crosslink under the same experimental conditions. The finding that cytosolic residues do not crosslink confirms that the copper chloride, which is membrane impermeant, is not artifactually gaining access to the cytosol under the conditions used. Moreover, we regret that our rationale for analyzing IpaC cysteine substitution derivatives with copper-induced crosslinking was unclear and have now provided a more extensive explanation of why this approach provides data that is informative and complementary to the data published in Russo et al., 2019 (lines 130-136).**

Fig. 1b: Nice scheme that helps with domain organization. Indicating the position of crucial mutants could be helpful for reader to directly localize them in protein context.

**We have added dots on the IpaC schematic to show the relative position of residues that were mutated (Fig 1b).**

Put a model in suppl fig. in main text. Probably needs revision with indicating single steps that could resemble conformational changes. Docking competent one: extracellularly accessible loop would need to go and bind Vim to open pore, it should block pore if inside pore?

**As suggested, we have moved the model to the main text (new Fig 5). We appreciate the reviewer’s suggestions but decided to not change the model. The diameter of the pore, as measured by osmoprotection, is not altered by the interaction of IpaC with intermediate filaments (Russo et al., 2016, *Nature Microbiology*), suggesting that the extracellularly accessible loop does not block the pore.**

• Figure 4 could be merged with Fig. 3-

**We thank the reviewer for the suggestion. Since the biological question that is addressed in Figure 4 differs substantially from that which is addressed in Figure 3, we have left them as independent figures.**

• Findings could be discussed more based on literature (25 references)

**We have included additional relevant literature references throughout the manuscript.**

• Paper could be written more concise

**We have made the writing more concise in those places where we felt that this did not affect its clarity.**

Reviewer #2: Line 163: “Thus, the formation of disulfide bonds at S17C, A38C, S63C, A106C, K350C, A353C, and likely some of the bonds formed at A363C, are accessible from the extracellular environment.” There is a problem with these experiments. The authors don’t know what percentage of IpaC detected in these experiments is actually part of an assembled translocation pore. Translocator proteins are exported at a low rate (leakage) before host cell contact, so IpaC detected in these experiments could be stuck to the outside and not properly inserted. They could also be inserted, but not part of a translocation pore. So, to my mind, the disulfide bond clearly occurs in the pore if there is an associated phenotype that is DSB-dependent (e.g. A38C and A353C), but the converse is less clear.

**We agree with the reviewer and have qualified our interpretation of these data in the text accordingly (lines 172-177). Moreover, over the course of these experiments, leakage of IpaC is not appreciable, as bacteria are resuspended in fresh media at the time of infection.**

Line 399 onward: Not sure what this is about. From the cited paper: “Given that PopDBpyFL efficiently interacts with PopB, the lack of increase in anisotropy was indicative of the presence of at least two or more PopDBpyFL subunits per PopB/PopD hetero-complex. Taken together, these results showed that PopD assembles homo-oligomeric structures in membranes, but when added together with PopB it forms hetero-oligomeric structures of yet undetermined size and stoichiometry.” I don’ think the data in the cited paper demonstrates an exact alternating PopB-PopD pattern, and therefore don’t conflict with the data from cell-based assays. Although the caveat noted by the authors, that in vitro systems can produce data that do not match data found cell-based systems, is valid.

**We agree with the reviewer and removed this topic of discussion.**

What the authors fail to mention is that in ref. 9, the authors demonstrate that using a disulfide bond to trap the PopD homodimer specifically interferes with host-cell sensing. The interpretation is that the pore has to undergo a conformational change to trigger effector secretion. This is obviously reminiscent of the model proposed here (and was derived by a similar methodology). I think this should be mentioned in the discussion (and perhaps the intro, line 86, could be modified to set this up). On the whole, the data support the findings in this manuscript, using a different bacterium, so this is a good thing.

**We regret that our discussion of these findings was not as clear as it could have been and now include more detailed discussion of the relevance of these published findings to the conclusions of this manuscript in both the Introduction and the Discussion (lines 93-94 and 410-413).**

Fig. 4a. Looks like more than 1bug/cell, so the images don’t really match up well with the quantitation data. Maybe a different set of images could illustrate this point better. Or is the average of 1bug/cell derived from 10 bugs attaching to 1, and 0 to 9 cells?

**Since under similar experimental conditions, *S. flexneri* infects approximately 50% of cells (Russo et. al. Nat. Micro. 2016), we would anticipate an average of 2-3 bacteria docked on each of 50% of the cells that are infected. We provide uncropped versions of the original cropped images, we believe that they reflect the rate of infection within an average image (Fig 4a).**

Line 288 “Since we found that intermolecular crosslinking of at the time …”, I would remove the “of”

**Changed as recommended.**

Line 475 should “adhesion” be “adhesin”?

**Changed as recommended.**

Reviewer #3: Minor comments
Line 288 : re-write sentence

**Changed as recommended.**

Line 351, there is some evidence for this, and some against. Include references here.

**Additional references have been added.**
